# An experimental EEG study of brain activities underlying the Autonomous Sensory Meridian Response

**DOI:** 10.1016/j.ibneur.2024.12.001

**Published:** 2024-12-07

**Authors:** Ali Mohammadi, Sahar Seifzadeh, Fatemeh Torkamani, Sina Salehi

**Affiliations:** a Shiraz Neuroscience Research Center, Shiraz University of Medical Sciences, Shiraz, Iran

**Keywords:** Autonomous sensory meridian response, ASMR, Electroencephalography, EEG

## Abstract

Autonomous Sensory Meridian Response (ASMR) is an audio-visual phenomenon that has recently become popular. Many people have reported experiencing a tingling-like sensation through their body while watching audio/video clips known as ASMR clips. People capable of having such experiences have also reported improved overall well-being and feeling relaxed. However, the neural activity underlying this phenomenon is not yet well-studied. The present study aims to investigate this issue using electroencephalography (EEG) employing an exploratory approach. We recorded resting-state EEGs from twelve participants before and after watching an ASMR clip and a control video clip. We divided the participants into two groups capable of experiencing ASMR tingling (ASMR group) and not capable of experiencing ASMR tingling (Non-ASMR group), by performing “Jenks Natural Breaks” clustering method on the results of a self-report questionnaire. We calculated the spectral power of EEG recording and compared the resulting values between the groups and sessions. We demonstrated a decline in the power of EEG activities in the delta frequency band in all regions of the brain and an increase in alpha activity in the occipital area of the brain and increases in beta oscillations was noted over the left fronto-temporal region of the brain among ASMR group. We did not observe similar results among the Non-ASMRs participants or among ASMRs in the control group.

## 1 Introduction

Some sounds like pressing a key on a keyboard, tapping on a mobile phone screen, or whispering in a quiet room could cause a pleasant sensation for some people, while the same sound could be irritating for others. A pleasant sensation upon hearing such sounds is known as Autonomous Sensory Meridian Response (ASMR), a non-scientific term suggested by Jennifer Allen in 2010 (Gallagher, 2019). ASMR was described as a tingling sensation in response to some audio-visual stimuli called “triggers” (Barratt et al., 2017, Barratt and Davis, 2015). ASMR triggers mainly exist in nature (e.g., blowing, rubbing, tapping) and in intimate relationships (e.g., whispering, gum chewing), which cultivate a peaceful and calm environment for the audience. Sensation usually starts from the head and neck and sometimes travels throughout the back and the rest of the body (Barratt & Davis, 2015; G. L. Poerio, 2016). Moreover, experiencing ASMR is associated with reduced heart rate and increased skin conductance level (G. L. Poerio et al., 2018). However, these experiences are usually unique to individuals, so that some people labeled as “Non-ASMR” never experience such feelings (G. L. Poerio, 2016; G. L. Poerio et al., 2018). Interestingly, it has been shown that ASMR and Non-ASMR individuals (groups) have different personality traits (B. K. Fredborg et al., 2017). In a study, the authors invited a large number of people to watch ASMR videos and asked them to report their experiences. Over half of the participants reported that they experienced ASMR (Barratt and Davis, 2015, Smith et al., 2017). This controversial audio-visual phenomenon has been known among non-academic communities since 2007 (Gallagher, 2019), and caught the eyes of mainstream media such as BBC News Magazine (Higham, 2014), the New York Times (Fairyington, 2014, Lauren Keiles, 2019) and the Washington Post (Gibson, 2014) in the past decade. The performers who developed these audio-visual videos are called “ASMRtists” and they usually share ASMR videos on social media such as YouTube or Instagram. Barratt and Davis (2015) have widely investigated the most popular triggers from an online questionnaire comprising 475 volunteer participants. They showed that the three most popular triggers were whispering (75 %), personal attention (69 %), and crisp sounds (64 %), respectively. Furthermore, Non-ASMR sounds (e.g., vacuum cleaner noise, airplane noise) have been compared with ASMR triggers, and the results showed that Non-ASMR sounds caused ASMR experience in less than 3 % of the participants.

In a study on the characteristics of sounds that trigger ASMR experience, the authors used a questionnaire completed by 130 ASMR-experienced participants. They found that lower-pitch complex sounds were the most effective sound feature. In contrast, background music suppressed the ASMR experience (Barratt et al., 2017). The reason that whispering is the most popular trigger could be that it is a sound between lovers in intimate relationships, or it might be remembered as maternal intimacy between a mother and a child (Andersen, 2015). Some people experienced this warm feeling from their childhood, for example, while having their haircut or being combed (G. L. Poerio, 2016; G. L. Poerio et al., 2018). This observation has guided the theory that ASMR might be associated with the act of grooming (Lochte et al., 2018; G. L. Poerio et al., 2018), while other studies suggested that ASMR-related experiences could be associated with mindfulness (Campo & Kehle, 2016; B. K. Fredborg et al., 2018). Physiological changes related to ASMR videos such as reduction in heart rate and an increase in skin conductance have been reported suggesting a calming effect and potential stress reduction (G. L. Poerio et al., 2018).

Research investigating brain activity associated with ASMR through fMRI has identified decreased connectivity in the default mode network (DMN) in individuals who experience ASMR compared to a control, non-ASMR group. These changes in functional connectivity were proposed to contribute to the unique sensory and emotional experiences specific to ASMR (Smith et al., 2017). Additional fMRI studies have shown that participants watching ASMR videos reported feelings of relaxation and tingling sensations, which were linked to increased activation in regions associated with reward and emotional arousal, specifically the nucleus accumbens, dorsal anterior cingulate cortex, and insula/IFG (Lochte et al., 2018, Sakurai et al., 2023). EEG studies exploring the ASMR experience have reported increased frontal alpha activity, as well as sensorimotor rhythm and gamma activity over the primary and secondary motor and somatosensory cortices (B. K. Fredborg et al., 2021), while other studies observed reduced alpha activity in the temporal region (Engelbregt et al., 2022). Another EEG study found decreased prefrontal delta power, increased alpha power in parietal, frontal, and temporal regions, increased low beta power in parietal and temporal regions, and decreased high beta power in parietal and occipital regions during ASMR experiences (Swart et al., 2022). Finally, ASMR combined with mental tasks was associated with increased occipital alpha activity on EEG (Inagaki & Ohta, 2022).

To this date, there are few EEG studies that aim to investigate the underlying neural activities associated with watching ASMR videos. This study follows an exploratory approach to expand the accumulating evidence of neural signature associated with ASMR experience in the brain.

## 2 Materials and methods

### 2.1 Participants and experimental design

The participants have been recruited among university students and all experiments were conducted in accordance with the Declaration of Helsinki and ethical guidelines for human subjects and approval of the Shiraz University of Medical Sciences ethical committee. All the procedures were carried out with adequate understanding and written consent of the human subjects. We instructed all the participants to rest enough the night before each session, don’t drink much coffee and keep their hair dry and clean to improve the quality of the EEG recordings. Two separate groups of people participated in this study. We asked the first group (n = 13, seven women and six men) aged 18–24 years (Mean±SD: 21 ± 1.95 years) to watch three different ASMR videos and rank their effect in terms of the tingling sensation they encountered while watching. The second group (n = 12, nine men and three women) aged 16–33 years (Mean±SD: 22.6 ± 3.9 years) participated in the EEG study. Neither group had encountered ASMR before. None of the participants had a history of neurological disease, psychiatric disorder, vision or hearing problems, or substance abuse. Three very popular ASMR videos with several triggers were selected from YouTube. These three ASMR videos were as follows: 1- ASMR Haircut Roleplay by Evan’s ASMR (evansasmr.com/youtube-archive/video-2017–08–26-asmr-haircut-roleplay/ - 23 minutes), 2- "ASMR Eye Exam Role Play *Whisper* ” by WhispersUnicorn (youtube.com/watch?v=MHwiO-jv8Gs - 19 minutes 41 seconds), and 3- "ASMR HIGH-SPEED TAPPING | Fast. Aggressive. No Mercy" by Asmr zeitgeist (youtube.com/watch?v=mRZn5-BfEl4 - 23 minutes 8 seconds). We asked the first group to watch these videos and rank them on a Likert scale (1−5) based on how much they experienced tingling sensations while watching the videos. The sum of the score for each video is used to rate them. The participants rated the third video as the most effective one (total score of respectively 41, 38 and 57, Table 1), which was also the most viewed on YouTube. Hence, we selected the third video to use as the ASMR stimuli in this study. This video contained some of the most common and effective ASMR triggers, e.g., whispering and tapping. Additionally, we chose a "TED Talk" video (ted.com/talks/sir_ken_robinson_bring_on_the_learning_revolution) as the control video. This video clip was 18 minutes long and contained none of the ASMR triggers reported.

**Table 1:** The results of *t*-test comparison between each pair of candidate video clips. The p-values are adjusted using the “Benjamini–Hochberg procedure”.

**Pair**	**P-Value**	**Effect Size (Cohen's d)**
**Video 1 VS. Video 2**	0.641	0.237
**Video 1 VS. Video 3**	0.053	−1.086
**Video 2 VS. Video 3**	0.002	−1.526

**Fig. 1:**
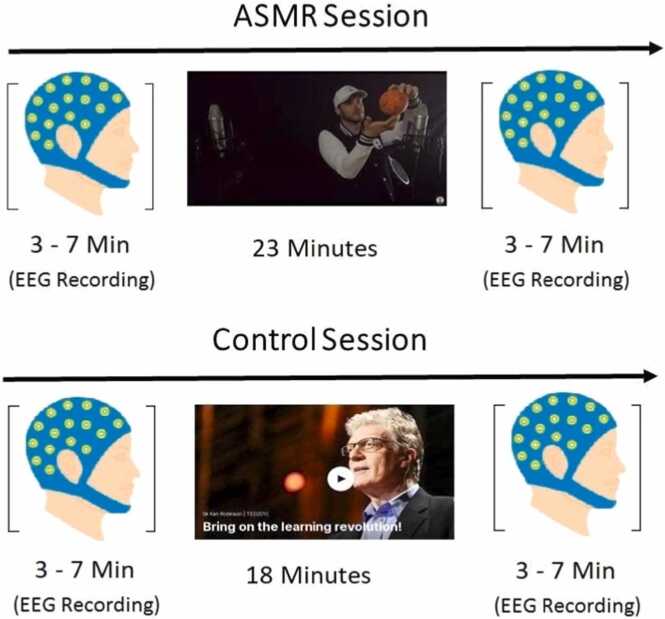
The schema of 2 experiment sessions for each subject.

We invited participants in the second group to partake in two separate EEG experiment sessions: an ASMR session and a control session, conducted on different days with the control session always following the ASMR session. Regrettably, four participants (2 males, 2 females) withdrew from the study after completing the ASMR session and did not participate in the control session. In the ASMR session, the involved participants watched the selected ASMR video, whereas in the control session, they watched a "Ted Talk" show. Participants sat on a comfortable chair 70 cm away from the 24-inch computer monitor in a dim-lit and quiet room. The audio was played using high-quality headphones. Resting-state EEG signals were recorded two times in each session, 3–7 minutes before and 3–7 minutes after watching the video (Fig. 1). At the beginning of each session, we instructed the participants to stay relaxed and minimize movements during EEG recordings.

### 2.2 Questionnaires

Before and after each ASMR and control session, we asked the participants to complete two self-report questionnaires to assess their emotional state and their responses to the ASMR video. The first questionnaire was the Persian translation of Positive and Negative Affect Schedule (PANAS) (Watson et al., 1988) used to evaluate emotional state. The validity and reliability of the Persian version of PANAS has been previously assessed in the Iranian population (Ahadi et al., 2018, Bakhshipour and Dezhkam, 2006). We designed another self-report questionnaire to evaluate the subjects' experience of tingling in six parts of the body, including scalp, neck, shoulders, lower back, arms, and legs while watching the ASMR video. This questionnaire includes six questions with five-point Likert scale responses ranging from "negligible or none" (1) to "extremely" (5).

### 2.3 EEG data acquisition and preprocessing

A 32-channel resting-state EEG was recorded using the ANT (ANT Neuro corp.) data acquisition device at a 625 Hz sampling rate. The impedance of all EEG electrodes was kept below 5KΩ throughout the recording. The raw EEG data for each participant, session, and trial were first re-referenced using the average of two electrodes, M1 and M2. The remaining 30 electrodes underwent a series of preprocessing steps to ensure the quality and reliability of the data for analysis. All EEG data were then imported to the EEGLAB toolbox (Delorme & Makeig, 2004) for further processing. We implemented a pipeline in MATLAB (ver. R2016b) using functions of the EEGLAB toolbox to perform preprocessing of the EEG data. We designed the data preprocessing pipeline to integrate manual intervention with Independent Component Analysis (ICA, Delorme et al., 2007; Makeig et al., 1995) application, aiming to minimize data exclusion while ensuring the integrity of the neural signals. A bandpass filter with a range of 1–70 Hz was applied to the data from the 30 electrodes to isolate the brain's electrical activity from potential low-frequency drift and high-frequency noise. The processed data were then subjected to a visual inspection to identify and reject segments containing abnormal or noisy data, primarily muscle artifacts. Following this, a two-round ICA process was employed:

*First Round of ICA*: This step did not involve the rejection of any components. Instead, it focused on identifying intervals of raw data that appeared in more than one component, indicative of noise or abnormal activity, which were then excluded. This approach aimed to enhance the quality of data for the subsequent ICA round.

*Second Round of ICA*: In this round, specific components associated with ocular-originated artifacts and noise affecting signals recorded at temporal electrodes (T7 and T8) were identified and rejected. Typically, one or two components were excluded based on their association with these artifacts.

Finally, for each subject, 90 seconds of noise-free EEG signal before the experiment and 90 seconds after (ASMR and control session) were selected for further analysis from all channels. The 90 seconds of post-preprocessing data for each session were divided into 30 non-overlapping segments of 3 seconds each. The EEG spectral power in four frequency bands (delta, theta, alpha, beta) was computed within these segments.

### 2.4 Statistical data analysis

a) PANAS Questionnaire: The PANAS questionnaire includes two sets of questions, negative and positive affect, and each set consists of 10 items. Each could be scored from 1 (Negligible or none) to 5 (extremely). The participants completed the questionnaire before and after watching the ASMR video. Scores across negative and positive effects and different groups were averaged separately. Differences in positive and negative scores for each group were compared using the *t*-test.
b) Self-Report ASMR Questionnaire: We used a self-report questionnaire to measure the sense of tingling in each participant by calculating sum of all self-reported scores for tingling sensation in individual body parts. In addition, we used the “Jenks Natural Breaks” classification method to divide the participants into two groups (Table 2), namely ASMRs (capable of experiencing ASMR) and Non-ASMRs (incapable of experiencing ASMR) based on this questionnaire.

**Table 2:** The results of the self-reported tingling sensations in various body parts, as noted by participants at the end of the ASMR session, in the order of the total tingling score defined as the sum of individual scores per participant. The intensity of the tingling sensation in each body part was measured on a Likert scale, employing descriptive terms for potential responses, ranging from "Negligible or none" (1) to "Slightly" (2), "Moderately" (3), "Substantially" (4), and "Extremely" (5). We applied the "Jenks Natural Breaks" method to the total tingling scores to divide participants into Non-ASMRs and ASMRs (denoted by red and green fonts, respectively).

**Subject #**	**Scalp**	**Neck**	**Shoulders**	**Lower Back**	**Arms**	**Legs**	**Total Tingling Score**
**01**	1	1	1	1	1	1	**6**
**02**	1	1	1	1	1	1	**6**
**03**	2	1	1	1	1	1	**7**
**04**	2	3	1	1	1	1	**9**
**05**	2	2	2	1	1	2	**10**
**06**	2	3	3	3	1	2	**14**
**07**	1	4	1	4	3	1	**14**
**08**	4	2	3	1	1	3	**14**
**09**	1	4	1	1	3	4	**14**
**10**	5	2	3	2	1	1	**14**
**11**	1	3	4	3	4	5	**20**
**12**	5	3	3	3	3	4	**21**
c) EEG analysis: The First 90 seconds of EEG signals were cut and divided into 30 non-overlapping epochs with three-second long epochs. The power spectral density for each epoch was estimated in the frequency bands delta (1–4 Hz), theta (4–8 Hz), alpha (8–12 Hz), and beta (12.5–30 Hz), by multi-taper frequency transformation using a Hanning window implemented in the Fieldtrip toolbox (Oostenveld et al., 2011). The values for each frequency band, subject, and session were normalized using z-scores to account for inter-individual variability. Differences between corresponding segments pre- and post-event were calculated for each subject and session. Averaging was performed across subjects for each group, session, trial, frequency band, electrode, and segment to maintain the relative values between different conditions. The differential values obtained from the normalization and pooling phase were subjected to *t*-test analysis against a null hypothesis value of zero. This step aimed to identify significant changes attributable to the experimental conditions. The results for each combination of group, session, and frequency band (with electrodes pooled together) underwent False Discovery Rate (FDR) correction using the “Benjamini–Hochberg procedure” to control for multiple comparisons (Glickman et al., 2014, Noble, 2009). We also calculated and reported the value of Cohen's d effect to quantify the magnitude of the observed differences (Cohen, 1988).

To evaluate changes over global areas we performed regional cluster analysis. We clustered electrodes in five distinct brain regions: Frontal (Fp1, Fpz, Fp2, F7, F3, Fz, F4, F8, FC5, FC1, FC2, FC6), Temporal (T7, T8), Central (C3, Cz, C4), Parietal (P7, P3, Pz, P4, P8, CP5, CP1, CP2, CP6), and Occipital (O1, Oz, O2, POz). The EEG power was averaged across all electrodes within each region for each frequency band, group, and session to compare pre- and post-ASMR regional activity (Barry & De Blasio, 2017). For the regional EEG power analysis, the average of the normalized power for each of the five brain regions (Frontal, Temporal, Central, Parietal, and Occipital) was calculated across all electrodes within each region for each frequency band, group, session, and segment. A non-parametric test was applied to evaluate whether the differences between pre- and post-ASMR regional EEG power were statistically significant across the different brain regions. The resulting p-values were subjected to FDR correction to account for multiple comparisons.

## 3 Results

### 3.1 Self-report questionnaire

Based on the self-report questionnaire described in the methods section, two groups were identified. The first group, ‘ASMRs’ (three women and four men), reported a high level of tingling sensation in different parts of the body (Mean±SD; 15.8 ± 3.2), and the second group, ‘Non-ASMRs’ (five men), reported negligible or no tingling sensation (Mean±SD; 7.6 ± 1.8).

### 3.2 PANAS questionnaire

The PANAS questionnaire revealed a significant decrease in negative feelings among ASMR group after watching the ASMR video (P = 0.02, Table 3)) whereas there were no significant changes in negative emotions among non-ASMR group (P = 0.46, Table 3). Additionally, no significant differences were observed between these two groups in terms of positive emotions (P > 0.7, Table 3).

**Table 3:** Comparison of the results from the PANAS questionnaire, reported by participants at the beginning and end of the ASMR session (pre- and post-watching the ASMR video), revealed significant insights. The PANAS questionnaire items are categorized into two sets: positive and negative. Participants responded on a Likert scale ranging from "negligible or none" (1) to "extremely" (5). The sum of the scores obtained for each item set was used for further analysis. Utilizing *t*-test analysis, we observed a significant decrease in negative emotions among ASMR participants at the session's end compared to its start.

Group	Emotion	Pre (SD)	Post (SD)	PValue	Effect Size (Cohen's d)
ASMR	+	33.00(6.5)	32.14(7.81)	0.795	0.102
ASMR	-	23.28(8.3)	14.57(4.5)	0.033	1.037
Non-ASMR	+	36.00(4.63)	34.60(5.9)	0.5	0.331
Non-ASMR	-	20.20(7.85)	17.80(4.5)	0.455	0.369

### 3.3 EEG data

**Fig. 2:**
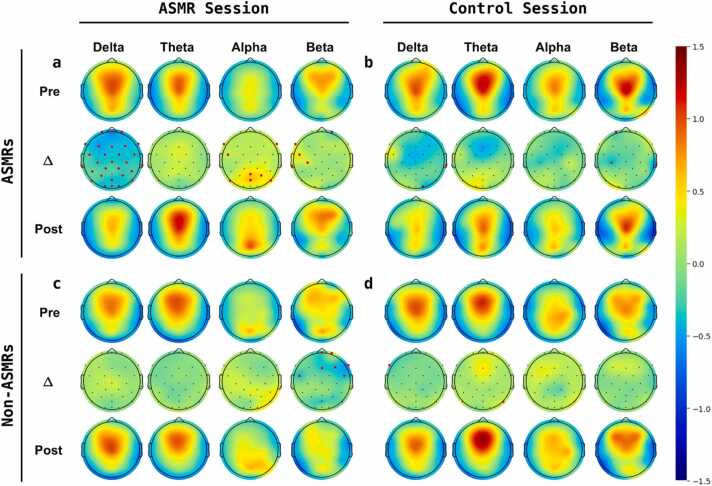
Topographically mapping of power-spectral analysis of the EEG signal in the different frequency bands. a) Average power spectral of ASMR group in the ASMR session shows significant changes in delta, alpha, and beta before and after the video. b) Average power spectral of ASMR group in the control session shows no significant changes before and after the video. c) Average power spectral of Non-ASMR group in the ASMR session shows a significant decrease in beta before and after the video. d) Average power spectral of Non-ASMR group in the control session shows no significant changes before and after the video.

**Fig. 3:**
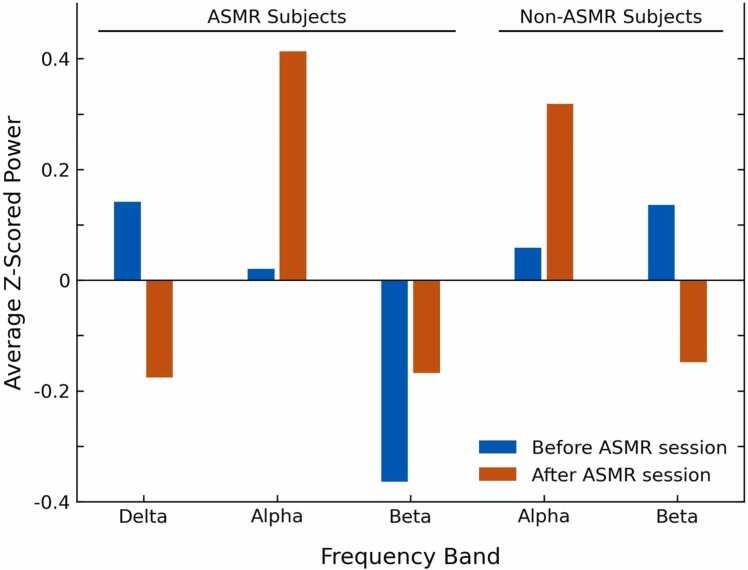
This graph demonstrates the averaged, z-scored EEG power in different frequency bands before (blue) and after (red) the ASMR sessions for the brain areas where the differences were significant (red dots in Fig. 2). The three bars Delta, Alpha and Beta on the left side of the graph showing the power difference for the ASMR experienced subjects and the two bars Alpha and Beta on the right side of the graph showing the power difference for NonASMR experienced subjects. Delta ASMR subjects: all electrodes except ‘Pz’; Alpha ASMR subjects: occipital area (‘P7’, ‘Pz’, ‘P4’, ‘P8’ and ‘POz’); Beta ASMR subjects: left parietal area (‘F7’, ‘FC5’, ‘T7’ and ‘C3’); Alphe NonASMR subjects: right occipital electrodes (‘CP2’, ‘CP6’, ‘P4’, ‘P8’ and ‘O2’); Beta NonASMR subjects: right frontal area (‘Fpz’, ‘Fp2’, ‘Fz’, ‘F4’, ‘F8’ and ‘FC6’).

Power spectral analysis of the EEG shows that resting-state activity significantly alters in delta, alpha, and beta frequency bands in ASMR group, and in alpha and beta frequency bands in Non-ASMR group after watching the ASMR video (Fig. 2, Fig. 3, Table 4, and Table 6). In the ASMR session of ASMR group, we observed a global decrease in the delta band oscillation, which was significant in almost all areas (-0.32 changes in averaged Z-scored power, P < 0.05, FDR-corrected, Table 4). A significant increase in the alpha band activity was found over the brain's occipital area (areas P7, Pz, P4, P8, and POz; 0.39 changes in averaged Z-scored power, P < 0.01, FDR-corrected, Table 4). A further increase in beta oscillations was noted over the left fronto-temporal region of the brain. (areas F7, FC5, T7, and C3; 0.2 changes in averaged Z-scored power, P < 0.01 FDR-corrected, Table 4). On the other hand, we found significant decrease beta activity over right frontal sites for Non-ASMR group in ASMR session (areas Fpz, Fp2, Fz, F4, F8, and FC6; −0.28 changes in averaged Z-scored power, P < 0.01, FDR-corrected, Table 6). An increase in occipital alpha activity has been observed in the ASMR session of Non-ASMR subjects, although it was not significant (areas CP2, CP6, P4, P8, and O2; 0.26 changes in averaged Z-scored power, P <0.1, Table 6). There was no other significant change observed in any of the groups after watching the control video (Fig. 2, Table 5, and Table 7).

**Table 4:** FDR corrected p-value (Cohen’s d effect size) for changes in power spectral in the alpha, beta, delta, and theta frequency band for ASMR group after watching ASMR Video.

Electrode	Delta	Theta	Alpha	Beta
Fp1	7.83E−04 (0.75)	4.86E−01 (0.18)	1.69E−01 (0.28)	9.27E−01 (0.04)
Fpz	6.78E−07 (1.39)	7.36E−01 (0.09)	1.09E−01 (0.36)	9.27E−01 (0.04)
Fp2	3.35E−05 (1.07)	8.88E−01 (0.03)	1.26E−01 (0.33)	1.63E−02 (0.63)
F7	3.21E−03 (0.62)	3.96E−01 (0.23)	2.55E−02 (0.58)	1.63E−02 (0.65)
F3	5.50E−04 (0.81)	3.96E−01 (0.26)	1.55E−01 (0.30)	9.82E−01 (0.01)
Fz	6.02E−03 (0.56)	3.96E−01 (0.35)	1.85E−01 (0.26)	6.56E−01 (0.18)
F4	2.03E−03 (0.68)	3.96E−01 (0.24)	1.26E−01 (0.34)	4.82E−01 (0.24)
F8	3.10E−04 (0.87)	9.84E−01 (0.00)	2.92E−03 (0.82)	1.93E−01 (0.36)
FC5	3.46E−03 (0.61)	3.96E−01 (0.33)	4.70E−02 (0.49)	3.82E−03 (0.81)
FC1	5.15E−03 (0.58)	3.96E−01 (0.31)	1.66E−01 (0.29)	9.27E−01 (0.08)
FC2	3.14E−03 (0.63)	3.96E−01 (0.36)	1.69E−01 (0.28)	9.27E−01 (0.04)
FC6	3.10E−04 (0.87)	8.88E−01 (0.03)	1.09E−01 (0.36)	7.48E−01 (0.16)
T7	6.59E−04 (0.77)	5.70E−01 (0.15)	5.91E−01 (0.10)	3.99E−02 (0.55)
C3	5.36E−03 (0.58)	4.86E−01 (0.18)	5.02E−01 (0.13)	4.56E−02 (0.52)
Cz	3.10E−02 (0.42)	3.96E−01 (0.26)	1.66E−01 (0.29)	9.27E−01 (0.10)
C4	2.40E−03 (0.66)	3.96E−01 (0.23)	6.26E−02 (0.42)	9.82E−01 (0.00)
T8	3.39E−05 (1.05)	3.96E−01 (0.35)	2.68E−01 (0.21)	5.29E−02 (0.50)
CP5	1.01E−02 (0.52)	3.96E−01 (0.25)	2.66E−01 (0.22)	5.51E−01 (0.22)
CP1	3.67E−02 (0.41)	3.96E−01 (0.30)	1.41E−01 (0.32)	9.27E−01 (0.08)
CP2	2.29E−02 (0.45)	3.96E−01 (0.23)	6.26E−02 (0.45)	9.27E−01 (0.04)
CP6	2.66E−03 (0.65)	5.70E−01 (0.14)	6.26E−02 (0.44)	9.82E−01 (0.01)
P7	6.59E−04 (0.77)	8.48E−01 (0.06)	4.54E−02 (0.50)	1.33E−01 (0.41)
P3	4.70E−02 (0.38)	3.96E−01 (0.29)	7.36E−02 (0.40)	9.27E−01 (0.09)
Pz	1.02E−01 (0.31)	3.96E−01 (0.21)	2.63E−02 (0.55)	9.27E−01 (0.06)
P4	7.61E−03 (0.54)	3.96E−01 (0.25)	1.81E−02 (0.63)	5.51E−01 (0.21)
P8	7.03E−03 (0.55)	6.18E−01 (0.13)	1.02E−02 (0.69)	9.27E−01 (0.04)
POz	2.95E−03 (0.64)	6.37E−01 (0.12)	2.63E−02 (0.56)	3.48E−01 (0.29)
O1	5.50E−04 (0.81)	3.96E−01 (0.21)	6.26E−02 (0.42)	1.93E−01 (0.36)
Oz	1.19E−03 (0.72)	3.96E−01 (0.23)	6.26E−02 (0.43)	9.27E−01 (0.12)
O2	6.11E−04 (0.79)	3.96E−01 (0.41)	6.26E−02 (0.43)	2.88E−01 (0.31)

**Table 5:** FDR corrected p-value (Cohen’s d effect size) for changes in power spectral in the alpha, beta, delta, and theta frequency band for ASMR group after watching the control Video.

Electrode	Delta	Theta	Alpha	Beta
Fp1	5.27E−01 (0.14)	4.50E−01 (0.24)	7.08E−01 (0.17)	1.40E−02 (0.64)
Fpz	3.34E−01 (0.23)	4.50E−01 (0.21)	6.88E−01 (0.19)	8.58E−01 (0.08)
Fp2	2.38E−01 (0.31)	4.50E−01 (0.23)	5.86E−01 (0.24)	7.71E−01 (0.12)
F7	3.32E−01 (0.25)	5.48E−01 (0.16)	9.82E−01 (0.01)	4.14E−01 (0.29)
F3	2.55E−01 (0.29)	2.62E−01 (0.36)	7.08E−01 (0.16)	5.16E−01 (0.20)
Fz	2.33E−01 (0.32)	1.96E−01 (0.45)	4.60E−01 (0.30)	5.46E−01 (0.19)
F4	1.07E−01 (0.44)	3.05E−01 (0.33)	4.60E−01 (0.37)	7.71E−01 (0.11)
F8	9.64E−02 (0.48)	4.50E−01 (0.20)	5.86E−01 (0.24)	8.38E−02 (0.49)
FC5	3.32E−01 (0.24)	4.50E−01 (0.19)	7.08E−01 (0.17)	3.43E−01 (0.35)
FC1	1.35E−01 (0.41)	2.62E−01 (0.39)	4.60E−01 (0.31)	4.14E−01 (0.30)
FC2	1.45E−01 (0.39)	2.62E−01 (0.36)	4.60E−01 (0.31)	4.86E−01 (0.23)
FC6	2.26E−01 (0.34)	4.50E−01 (0.19)	7.78E−01 (0.13)	9.92E−01 (0.02)
T7	7.81E−02 (0.52)	6.56E−01 (0.12)	4.60E−01 (0.33)	4.14E−01 (0.29)
C3	1.45E−01 (0.39)	4.50E−01 (0.20)	4.60E−01 (0.37)	1.32E−02 (0.68)
Cz	2.33E−01 (0.32)	4.50E−01 (0.22)	5.86E−01 (0.23)	4.53E−01 (0.27)
C4	3.32E−01 (0.23)	9.32E−01 (0.02)	6.29E−01 (0.21)	8.58E−01 (0.09)
T8	1.31E−02 (0.68)	7.35E−01 (0.09)	4.60E−01 (0.42)	6.81E−08 (1.55)
CP5	3.80E−01 (0.20)	5.48E−01 (0.16)	7.78E−01 (0.10)	9.92E−01 (0.00)
CP1	3.32E−01 (0.25)	7.49E−01 (0.07)	9.22E−01 (0.04)	5.16E−01 (0.21)
CP2	3.80E−01 (0.20)	6.56E−01 (0.11)	7.86E−01 (0.10)	4.86E−01 (0.24)
CP6	3.32E−01 (0.25)	7.49E−01 (0.07)	8.04E−01 (0.09)	9.92E−01 (0.01)
P7	7.90E−01 (0.06)	1.96E−01 (0.51)	7.78E−01 (0.12)	4.88E−01 (0.22)
P3	7.90E−01 (0.06)	3.79E−01 (0.29)	7.78E−01 (0.13)	9.92E−01 (0.01)
Pz	5.59E−01 (0.12)	4.50E−01 (0.25)	9.82E−01 (0.01)	9.92E−01 (0.04)
P4	3.80E−01 (0.21)	4.50E−01 (0.23)	7.08E−01 (0.16)	7.00E−01 (0.14)
P8	9.64E−02 (0.47)	6.56E−01 (0.11)	9.82E−01 (0.00)	5.46E−01 (0.18)
POz	5.59E−01 (0.13)	3.79E−01 (0.30)	9.15E−01 (0.05)	9.92E−01 (0.02)
O1	5.05E−01 (0.15)	1.96E−01 (0.47)	7.78E−01 (0.11)	8.47E−02 (0.48)
Oz	8.31E−01 (0.04)	2.62E−01 (0.38)	9.15E−01 (0.06)	9.92E−01 (0.00)
O2	6.11E−03 (0.78)	9.23E−01 (0.02)	4.77E−01 (0.29)	4.86E−01 (0.23)

**Table 6:** FDR corrected p-value (Cohen’s d effect size) for changes in power spectral in the alpha, beta, delta, and theta frequency band for Non-ASMR group after watching ASMR Video.

Electrode	Delta	Theta	Alpha	Beta
Fp1	9.40E−01 (0.12)	6.31E−01 (0.33)	7.68E−01 (0.09)	8.29E−02 (0.40)
Fpz	9.40E−01 (0.08)	6.31E−01 (0.32)	9.75E−01 (0.01)	3.84E−02 (0.51)
Fp2	9.67E−01 (0.02)	8.73E−01 (0.04)	8.46E−01 (0.06)	2.58E−02 (0.58)
F7	9.40E−01 (0.25)	6.31E−01 (0.29)	7.08E−02 (0.51)	3.80E−01 (0.21)
F3	9.40E−01 (0.11)	8.47E−01 (0.16)	5.50E−01 (0.19)	1.80E−01 (0.31)
Fz	9.67E−01 (0.02)	8.73E−01 (0.05)	8.46E−01 (0.07)	1.55E−02 (0.64)
F4	9.40E−01 (0.09)	8.73E−01 (0.09)	2.40E−01 (0.32)	3.14E−03 (0.77)
F8	9.67E−01 (0.01)	8.73E−01 (0.10)	7.68E−01 (0.12)	2.87E−02 (0.56)
FC5	9.40E−01 (0.17)	6.31E−01 (0.33)	7.08E−02 (0.52)	3.84E−02 (0.51)
FC1	9.40E−01 (0.19)	8.73E−01 (0.07)	2.57E−01 (0.30)	2.78E−01 (0.26)
FC2	9.40E−01 (0.08)	8.73E−01 (0.04)	9.24E−01 (0.02)	6.80E−02 (0.44)
FC6	9.73E−01 (0.01)	8.73E−01 (0.05)	8.95E−01 (0.05)	3.14E−03 (0.80)
T7	9.67E−01 (0.03)	9.26E−01 (0.02)	1.98E−01 (0.38)	6.30E−01 (0.12)
C3	9.40E−01 (0.18)	8.47E−01 (0.16)	2.40E−01 (0.35)	9.54E−01 (0.01)
Cz	9.40E−01 (0.21)	8.73E−01 (0.07)	3.36E−01 (0.25)	3.05E−01 (0.24)
C4	9.40E−01 (0.09)	8.73E−01 (0.08)	7.61E−01 (0.14)	1.29E−01 (0.36)
T8	9.40E−01 (0.09)	6.31E−01 (0.43)	5.03E−02 (0.63)	6.11E−01 (0.13)
CP5	9.40E−01 (0.23)	8.73E−01 (0.05)	2.40E−01 (0.32)	8.82E−01 (0.03)
CP1	9.40E−01 (0.14)	8.47E−01 (0.19)	2.57E−01 (0.29)	8.56E−01 (0.06)
CP2	9.40E−01 (0.16)	8.47E−01 (0.16)	2.40E−01 (0.34)	8.71E−01 (0.04)
CP6	9.67E−01 (0.05)	8.73E−01 (0.07)	7.08E−02 (0.49)	8.29E−02 (0.40)
P7	9.40E−01 (0.07)	8.47E−01 (0.18)	7.68E−01 (0.12)	2.78E−01 (0.25)
P3	9.67E−01 (0.04)	8.73E−01 (0.12)	7.68E−01 (0.09)	2.00E−01 (0.30)
Pz	9.40E−01 (0.07)	8.73E−01 (0.14)	3.36E−01 (0.25)	4.32E−01 (0.19)
P4	9.40E−01 (0.07)	8.73E−01 (0.04)	2.40E−01 (0.31)	5.39E−02 (0.48)
P8	9.40E−01 (0.07)	8.73E−01 (0.12)	1.49E−01 (0.42)	1.66E−01 (0.33)
POz	9.40E−01 (0.14)	8.47E−01 (0.16)	9.09E−01 (0.04)	8.29E−02 (0.40)
O1	9.40E−01 (0.16)	8.47E−01 (0.22)	7.68E−01 (0.10)	6.80E−02 (0.44)
Oz	9.40E−01 (0.21)	8.47E−01 (0.17)	7.68E−01 (0.11)	8.56E−01 (0.06)
O2	9.40E−01 (0.08)	6.31E−01 (0.29)	7.08E−02 (0.50)	8.56E−01 (0.05)

**Table 7:** FDR corrected p-value (Cohen’s d effect size) for changes in power spectral in the alpha, beta, delta, and theta frequency band for Non-ASMR group after watching the control Video.

Electrode	Delta	Theta	Alpha	Beta
Fp1	2.12E−01 (0.48)	2.91E−01 (0.35)	8.26E−01 (0.05)	9.06E−01 (0.11)
Fpz	2.84E−01 (0.40)	2.91E−01 (0.32)	5.15E−01 (0.16)	9.06E−01 (0.10)
Fp2	9.12E−01 (0.14)	2.91E−01 (0.39)	4.36E−01 (0.23)	9.06E−01 (0.09)
F7	2.35E−02 (0.68)	4.39E−01 (0.20)	3.60E−01 (0.31)	2.39E−01 (0.40)
F3	9.12E−01 (0.15)	3.67E−01 (0.24)	9.56E−02 (0.54)	1.44E−01 (0.50)
Fz	9.12E−01 (0.09)	2.91E−01 (0.44)	2.38E−01 (0.41)	2.39E−01 (0.39)
F4	9.12E−01 (0.15)	2.91E−01 (0.30)	4.75E−01 (0.21)	9.06E−01 (0.19)
F8	9.12E−01 (0.09)	3.67E−01 (0.24)	3.48E−01 (0.34)	1.44E−01 (0.48)
FC5	6.45E−01 (0.30)	5.56E−01 (0.15)	9.56E−02 (0.54)	9.06E−01 (0.17)
FC1	9.12E−01 (0.10)	2.91E−01 (0.35)	2.38E−01 (0.39)	9.06E−01 (0.09)
FC2	9.12E−01 (0.10)	2.91E−01 (0.31)	5.75E−01 (0.13)	9.06E−01 (0.14)
FC6	6.45E−01 (0.29)	3.67E−01 (0.25)	3.69E−01 (0.27)	5.27E−01 (0.31)
T7	9.68E−01 (0.01)	3.67E−01 (0.26)	4.81E−01 (0.18)	9.02E−01 (0.21)
C3	9.12E−01 (0.13)	8.24E−01 (0.05)	3.81E−01 (0.26)	9.06E−01 (0.14)
Cz	9.12E−01 (0.08)	3.67E−01 (0.26)	8.42E−01 (0.04)	9.06E−01 (0.06)
C4	9.12E−01 (0.17)	5.56E−01 (0.15)	7.57E−01 (0.08)	9.06E−01 (0.13)
T8	9.68E−01 (0.03)	2.91E−01 (0.31)	5.75E−01 (0.13)	1.02E−01 (0.58)
CP5	9.68E−01 (0.03)	5.40E−01 (0.17)	4.33E−01 (0.24)	9.06E−01 (0.02)
CP1	9.68E−01 (0.01)	8.11E−01 (0.06)	4.81E−01 (0.18)	9.06E−01 (0.07)
CP2	9.12E−01 (0.18)	9.46E−01 (0.02)	3.60E−01 (0.30)	9.02E−01 (0.21)
CP6	9.68E−01 (0.04)	3.67E−01 (0.24)	3.60E−01 (0.30)	9.06E−01 (0.04)
P7	2.84E−01 (0.41)	2.91E−01 (0.32)	3.60E−01 (0.28)	9.06E−01 (0.03)
P3	9.12E−01 (0.08)	6.41E−01 (0.11)	8.26E−01 (0.05)	9.06E−01 (0.03)
Pz	9.68E−01 (0.03)	7.41E−01 (0.09)	5.75E−01 (0.14)	9.06E−01 (0.15)
P4	9.63E−01 (0.06)	4.31E−01 (0.21)	6.11E−01 (0.12)	9.06E−01 (0.04)
P8	6.50E−01 (0.27)	2.85E−02 (0.67)	2.38E−01 (0.42)	9.06E−01 (0.12)
POz	9.12E−01 (0.11)	6.17E−01 (0.13)	4.81E−01 (0.18)	9.06E−01 (0.08)
O1	9.12E−01 (0.12)	6.17E−01 (0.13)	4.81E−01 (0.19)	9.02E−01 (0.22)
Oz	9.12E−01 (0.10)	9.46E−01 (0.01)	4.81E−01 (0.19)	9.06E−01 (0.02)
O2	9.68E−01 (0.01)	2.91E−01 (0.32)	3.60E−01 (0.28)	9.06E−01 (0.04)

**Fig. 4:**
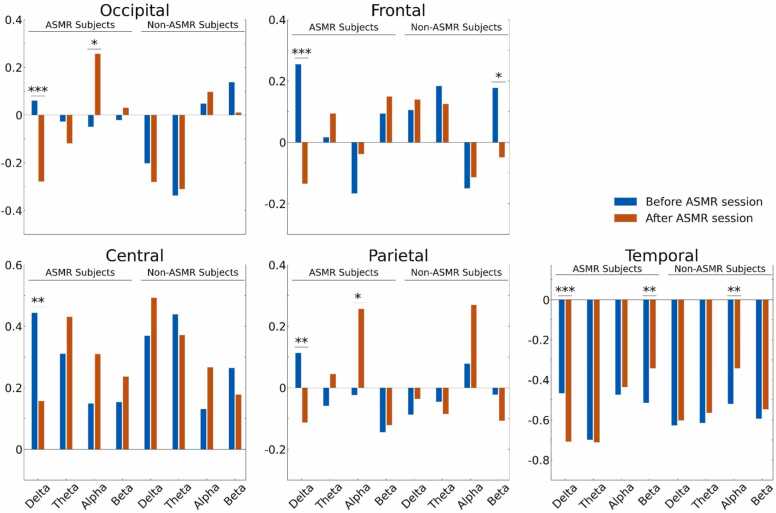
The averaged, z-scored EEG power in different frequency bands before (blue) and after (red) the ASMR sessions in different brain regions. Significant differences have been marked by ‘* ’ (* < 0.05, ** < 0.01, and *** < 0.001, FDR-corrected).

To evaluate changes over global areas we performed regional cluster analysis across five brain regions: Frontal, Temporal, Central, Parietal, and Occipital. The result of the regional EEG analysis was in line with the analysis of EEG data across individual electrodes. The regional analysis (Fig. 4) revealed a significant decrease in Delta band power in all regions, a significant increase in Alpha band power in Occipital and Parietal regions, and a significant increase in Beta band power in the Temporal region of the ASMR group after watching the ASMR video (Fig. 4 and Table 8). In contrast, the Non-ASMR group showed a significant increase in Alpha power in the Temporal region and a significant decline in Beta band power in the Frontal region after watching the ASMR video (Fig. 4 and Table 10). In the control session, the ASMR group showed a significant decrease in Beta and Delta band power in the Temporal region after watching the control video (Table 9) while Non-ASMR group showed no significant change in any region after watching the control video (Table 11).

**Table 8:** FDR corrected p-value (Cohen’s d effect size) for changes in average power of the alpha, beta, delta, and theta frequency band for ASMR group after watching the ASMR Video in different brain regions.

Brain Region	Alpha	Beta	Delta	Theta
Central	1.26E−01 (0.31)	6.28E−01 (0.21)	5.65E−03 (0.56)	2.73E−01 (0.24)
Frontal	6.00E−02 (0.40)	6.28E−01 (0.16)	7.07E−05 (0.91)	2.73E−01 (0.23)
Occipital	2.68E−02 (0.50)	6.28E−01 (0.12)	1.52E−04 (0.83)	2.73E−01 (0.24)
Parietal	2.68E−02 (0.55)	6.32E−01 (0.09)	6.18E−03 (0.54)	2.73E−01 (0.25)
Temporal	3.18E−01 (0.19)	7.99E−03 (0.64)	1.11E−05 (1.07)	6.17E−01 (0.09)

**Table 9:** FDR corrected p-value (Cohen’s d effect size) for changes in average power of the alpha, beta, delta, and theta frequency band for ASMR group after watching the control Video in different brain regions.

Brain Region	Alpha	Beta	Delta	Theta
Central	6.27E−01 (0.16)	9.88E−02 (0.39)	1.48E−01 (0.32)	4.63E−01 (0.16)
Frontal	3.80E−01 (0.27)	6.90E−01 (0.15)	1.48E−01 (0.34)	2.15E−01 (0.32)
Occipital	9.87E−01 (0.04)	7.44E−01 (0.06)	3.10E−01 (0.19)	2.15E−01 (0.34)
Parietal	9.87E−01 (0.00)	7.44E−01 (0.09)	2.94E−01 (0.22)	3.75E−01 (0.23)
Temporal	1.65E−01 (0.41)	1.23E−04 (0.91)	2.52E−03 (0.71)	4.63E−01 (0.14)

**Table 10:** FDR corrected p-value (Cohen’s d effect size) for changes in average power of the alpha, beta, delta, and theta frequency band for Non-ASMR group after watching the ASMR Video in different brain regions.

Brain Region	Alpha	Beta	Delta	Theta
Central	2.06E−01 (0.29)	2.93E−01 (0.22)	7.02E−01 (0.18)	6.71E−01 (0.11)
Frontal	6.08E−01 (0.13)	2.67E−02 (0.55)	7.02E−01 (0.07)	6.71E−01 (0.15)
Occipital	6.17E−01 (0.09)	2.47E−01 (0.29)	7.02E−01 (0.16)	6.71E−01 (0.09)
Parietal	1.21E−01 (0.38)	2.47E−01 (0.27)	7.02E−01 (0.11)	6.71E−01 (0.08)
Temporal	6.05E−03 (0.65)	3.83E−01 (0.16)	7.02E−01 (0.07)	6.71E−01 (0.23)

**Table 11:** FDR corrected p-value (Cohen’s d effect size) for changes in average power of the alpha, beta, delta, and theta frequency band for Non-ASMR group after watching the control Video in different brain regions.

Brain Region	Alpha	Beta	Delta	Theta
Central	5.62E−01 (0.12)	9.19E−01 (0.12)	9.69E−01 (0.13)	4.06E−01 (0.18)
Frontal	2.62E−01 (0.37)	9.19E−01 (0.02)	8.83E−01 (0.25)	1.38E−01 (0.36)
Occipital	5.07E−01 (0.24)	9.19E−01 (0.08)	9.69E−01 (0.01)	5.93E−01 (0.10)
Parietal	5.62E−01 (0.11)	9.19E−01 (0.06)	9.69E−01 (0.01)	4.06E−01 (0.18)
Temporal	5.50E−01 (0.18)	8.74E−02 (0.46)	9.69E−01 (0.03)	1.38E−01 (0.39)

## 4 Discussion

We recorded EEG data from human subjects before and after they viewed ASMR and control videos. Besides, we asked participants to complete a self-report questionnaire about their experience of ASMR video. Based on the self-report questionnaire result, we found that our participants fell into two groups: ASMR (capable of experiencing ASMR sensation) and Non-ASMR group (incapable of experiencing ASMR sensation). In the ASMR session of ASMR group, we found a global decrease of delta activity, especially in the frontal area, an occipital increase of alpha activity and increases in beta oscillations was noted over the left fronto-temporal region of the brain. No statistically significant change in brain activity was observed in other conditions (ASMR group in the control session, Non-ASMR subjects in ASMR, and control sessions).

Several findings from electrophysiological recording in anesthetized and freely moving animals support that slow-wave (SW; delta and theta) EEG activity originates in deeper brain regions such as the ventral tegmental area (Grace, 1995), ventral pallidum (Lavin & Grace, 1996), and the nucleus accumbens (Leung & Yim, 1993) involved in the brain reward system. This system is now believed to deal not only with rewards but salience detection (Gray, 1999, Knyazev, 2007, Miskovic et al., 2010). Delta oscillations are also found in the frontal and cingulate cortex (Harmony, 2013, Knyazev, 2012, Murphy et al., 2009). The observed enhancement of the delta activity in the so-called P300 experimental paradigm (go/no-go task) confirms the dominant role of delta oscillatory system in salience detection and suppression of interference during attention (Basar-Eroglu et al., 1992, Herrmann et al., 2016). We found a global decrease of delta activity in ASMR sessions of ASMR group, which could imply a lack of focused attention and increased relaxation in response to ASMR stimuli. Fulfillment of relaxation is correlated with decrements of delta power as assessed by self-report questionnaires (Teplan et al., 2014). However, in an fMRI study, participants who experienced ASMR showed significant activation in regions associated with both reward (NAcc) and emotional arousal (dACC and Insula/IFG), which is linked to the generation of delta oscillation in the brain (Lochte et al., 2018, Sakurai et al., 2023).

Second observation an increase in occipital alpha activity in the ASMR group after watching the ASMR video, consistent with findings from two recent ASMR studies (Inagaki and Ohta, 2022, Sakurai et al., 2023). Another study reported increased fronto-parietal alpha activity (B. K. Fredborg et al., 2021), while a separate study observed contrasting results, reporting a reduction in alpha activity (Engelbregt et al., 2022). Alpha oscillations in EEG are known to modulate during various cognitive processes, including sensory stimulation (Schürmann & Basar, 2001), memory (Klimesch, 1997), and attentional processes (Hanslmayr et al., 2011). Alpha oscillations have shown an inhibitory effect on cognitive performance, likely suppressing task-irrelevant cortical areas (Basar, 2012, Herrmann et al., 2016, Jensen and Mazaheri, 2010, Klimesch, 2012). Alpha EEG activity has also been positively associated with levels of drowsiness (Cantero and Atienza, 2000, Correa et al., 2014, Kartsch et al., 2017, Pal et al., 2008). Since our participants were not engaged in any task, and we recorded resting-state EEG, this increase in alpha activity could indicate a heightened level of drowsiness or relaxation. It is noteworthy that we compared resting-state brain activity before and after watching an ASMR video to assess any stable, persistent effects of ASMR on the brain, while most other studies recorded brain activity during the ASMR experience itself. This methodological distinction may contribute to the differences observed in our findings.

The last significant finding in the ASMR sessions of the ASMR group was increased left fronto-temporal beta power was inline with the recent study (Engelbregt et al., 2022). Fronto-parietal beta band modulation in human EEG was mainly observed when the participants performed sensorimotor tasks (Kilavik et al., 2013, Neuper and Pfurtscheller, 2001). Beta oscillation plays a role in the pathophysiology of movement disorders such as Parkinson’s disease, and increased beta activity is associated with worsening of voluntary movement and bradykinesia symptoms (Neumann et al., 2016, Petersson et al., 2020, Steiner et al., 2017). Growing evidence suggests that the beta band activity is involved in cognitive and sensorimotor tasks, and its increased activity reflects that the current sensorimotor state is expected to maintain in due course (Engel and Fries, 2010, Herrmann et al., 2016) or signals the stopping of action (Hannah & Aron, 2021). Thus, our finding of beta modulation suggests the participants’ motionlessness during the task and their inertia to remain still. Another possibility of increased beta activity over the sensorimotor area is related to experiencing ASMR tingling (B. K. Fredborg et al., 2021).

Taken together, our findings of decreasing delta, increased occipital alpha, and temporal beta indicated increased relaxation and calmness in the participants. These changes were specific, and none of the changes observed in the ASMR session of the ASMR group happened in other conditions. Above all, ASMR could be an inexpensive, portable, and effective supplementary therapy that is open to scientific investigation.

## CRediT authorship contribution statement

**Fatemeh Torkamani:** Methodology, Investigation, Conceptualization. **Sina Salehi:** Writing – original draft, Validation, Supervision, Software, Resources, Project administration, Methodology, Investigation, Funding acquisition, Formal analysis, Data curation, Conceptualization. **Ali Mohammadi:** Writing – original draft, Visualization, Validation, Software, Methodology, Investigation, Formal analysis, Data curation, Conceptualization. **Sahar Seifzadeh:** Writing – original draft, Methodology, Investigation, Conceptualization.

## Declaration of Competing Interest

None.

## Data Availability

The data that support the findings of this study are available on request from the corresponding author. The data are not publicly available due to privacy and ethical restrictions.
